# Fluorine-engineered thienyl-fused non-fullerene acceptors based on as-indacene for high-performance organic solar cells: a DFT study

**DOI:** 10.1039/d6ra05612a

**Published:** 2026-08-25

**Authors:** Sandeep Kumar, Anjali Singh, Mirtunjai Mishra, Amarjeet Yadav

**Affiliations:** a Department of Physics, Siddharth University Kapilvastu Siddharthnagar Uttar Pradesh 272202 India amarbhu1@yahoo.in

## Abstract

The growing demand for sustainable energy has driven the development of high-performance organic solar cells (OSCs). In this work, a series of donor–π–acceptor molecules were designed using 1,1,6,6-tetramethyl-*as*-indacene as the donor, furan as the π-spacer, and thienyl-fused IC derivatives as acceptors. Structural tuning was achieved through positional fluorine substitution and variation of the thienyl unit. Density functional theory (DFT) and time-dependent DFT (TD-DFT) calculations were employed to investigate electronic, optical, and photovoltaic properties. The designed molecules exhibit reduced HOMO–LUMO energy gaps (by ∼0.12 eV) relative to the reference, indicating enhanced charge-transfer characteristics. Fluorine substitution increases dipole moments, suggesting improved molecular stability. Additionally, strong absorption in the red region (780–820 nm) in chlorobenzene indicates enhanced light-harvesting ability. These findings demonstrate that fluorination and acceptor engineering effectively modulate optoelectronic properties, highlighting the potential of these materials for high-efficiency OSC applications.

## Abstract 2

The growing demand for sustainable energy has driven the development of high-performance organic solar cells (OSCs).

## Graphical abstract

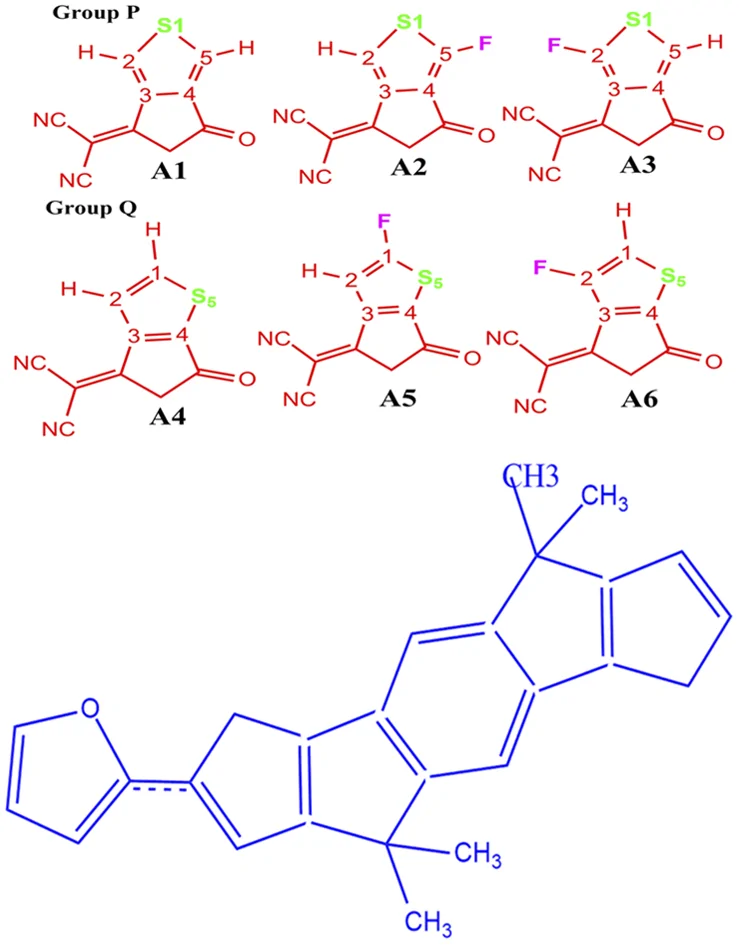

## 1. Introduction

The rapid depletion of natural resources, coupled with increasing global energy demand, has intensified the need for sustainable and environmentally friendly energy solutions.^1–4^ Conventional energy sources not only contribute to resource exhaustion but also lead to severe environmental pollution. In this context, renewable energy technologies have emerged as viable alternatives, among which solar energy stands out as one of the most promising and abundant sources.^5–7^

Among various photovoltaic technologies, organic solar cells (OSCs) have attracted significant attention due to their advantages, including low cost, mechanical flexibility, lightweight nature, and ease of fabrication.^8–11^ Typically, an OSC consists of a donor (D) and an acceptor (A) material, where efficient charge separation occurs at the donor–acceptor interface.^12^ To further enhance charge transfer efficiency, a π-conjugated spacer is often introduced between the donor and acceptor units, leading to a donor–π–acceptor (D–π–A) architecture.^13^

The design of high-performance OSC materials requires careful tuning of the molecular structure to achieve optimal band gaps that align with the visible and near-infrared (NIR) regions, where solar photon absorption is maximized. Additionally, functionalization with electron-withdrawing groups can significantly improve charge transfer efficiency and, consequently, the power conversion efficiency (PCE) of the device.^14–16^ Initially, fullerene and its derivatives were widely used as acceptor materials due to their high electron mobility and excellent electron-accepting ability.^17^ However, their limitations, such as weak absorption in the visible region, limited tunability, and morphological instability, have led to the development of non-fullerene acceptors (NFAs). NFAs offer several advantages, including tunable energy levels, strong absorption, and improved device stability, making them highly promising for next-generation OSCs.^18–20^ Recent advancements have demonstrated that NFA-based OSCs can achieve power conversion efficiencies exceeding 19%.^21,22^

Recent advances in non-fullerene acceptor (NFA) molecular engineering have demonstrated that precise control over terminal groups, molecular asymmetry, halogen substitution, and intermolecular packing can substantially influence the photovoltaic properties of organic solar cells (OSCs). For example, multiple-asymmetric molecular engineering through controlled modulation of fluorinated and chlorinated terminal groups has recently produced selenium-containing acceptors with a power conversion efficiency (PCE) of 19.32%, which was attributed to favorable molecular packing, enhanced crystallinity, charge transport, and reduced non-radiative energy loss.^23^ More recently, hetero-fluorinated/brominated asymmetric terminal engineering has been reported to deliver a binary OSC efficiency of 20.3%, highlighting the effectiveness of carefully controlling the number and position of halogen substituents in acceptor molecules.^24^ In another recent study, asymmetric acceptor engineering involving unidirectional terminal π-extension and optimization of alkyl branching sites resulted in a binary PCE of 20.7%, further demonstrating the importance of molecular asymmetry and structural organization in minimizing energy losses.^25^ In parallel with molecular design, recent studies have emphasized the critical roles of interfacial engineering, processing conditions, and thin-film morphology in determining the final device performance of OSCs. Self-assembled monolayers have been investigated as efficient hole-transport and interfacial layers, although their aggregation behavior can affect the formation of uniform films and consequently charge extraction.^26^ Environmentally friendly co-solvent strategies have recently been demonstrated to suppress such aggregation and improve the quality of interfacial layers,^27^ while non-halogenated co-solvent engineering has been shown to regulate crystallization kinetics, molecular packing, and nanoscale phase separation, enabling OSC efficiencies approaching 20%.^28^ Collectively, these recent developments demonstrate that high-performance OSCs arise from the combined optimization of molecular electronic structure, terminal-group engineering, intermolecular interactions, morphology, interfaces, and processing conditions. Therefore, systematic molecular-level investigation of fluorination and thienyl positional isomerism, as undertaken in the present work, provides a useful computational approach for identifying structure–property relationships in emerging NFA systems.

In this context, fused-ring electron acceptors (FREAs) have emerged as an important class of NFAs due to their enhanced optoelectronic properties and structural versatility. Representative examples include 1-dicyanomethylene-3-indanone (IC), diketopyrrolopyrrole (DPP), malononitrile, and rhodamine-based systems. Among these, thienyl-fused IC derivatives are particularly attractive, as their optoelectronic properties can be effectively tuned by altering the position of the thienyl unit.^29,30^ Furthermore, halogen substitution at different positions can significantly influence intermolecular interactions, including van der Waals forces, as well as electronic distribution and molecular stability, enabling rational design of high-performance materials.^30^ Recently, Taouali *et al.* (2025) reported a series of phenothiazine-based D–π–A molecules (M1–M6) incorporating thienyl-fused IC acceptors, exhibiting energy gaps in the range of 2.14–2.29 eV and open-circuit voltages around 1.69 V with PBDB-T as the donor.^31^ Such studies highlight the effectiveness of end-group engineering and computational design strategies in optimising the electronic properties and performance of OSC materials.^32,33^

Inspired by these advancements, the present work focuses on the design of novel D–π–A molecules using 1,1,6,6-tetramethyl-*as*-indacene as the donor unit, owing to its rigid and planar structure, strong π–π stacking, and favorable charge transport characteristics. A furan unit is employed as the π-spacer, while thienyl-fused IC derivatives are used as acceptor units.^34,35^

Six new molecules (D1–D6) were designed by introducing fluorine atoms at different positions on the thienyl-fused IC acceptors, categorised into two groups (P and Q) based on the positional variation of the thienyl unit. This structural modification strategy enables systematic investigation of the effects of fluorination and positional isomerism on the optoelectronic and photovoltaic properties of the molecules. The designed systems were comprehensively analysed using density functional theory (DFT) and time-dependent DFT (TD-DFT) calculations to evaluate their structural, electronic, optical, and photovoltaic properties. This study aims to provide deeper insights into structure–property relationships and to propose efficient molecular candidates for next-generation organic solar cell applications.

## 2. Computational technique

All quantum chemical calculations were performed using the Gaussian 16 program package.^36^ Density functional theory (DFT) and time-dependent density functional theory (TD-DFT) methods were employed to investigate the ground- and excited-state properties of the designed molecules (D1–D6). The geometries of all molecules were fully optimized at the B3LYP/6-311G(d,p) level of theory without symmetry constraints.^37,38^ The B3LYP functional was selected due to its proven reliability in predicting the electronic structure and excitation energies of organic systems.^39,40^

TD-DFT calculations were subsequently performed at the same level to determine the vertical excitation energies, oscillator strengths, and corresponding absorption wavelengths. B3LYP was selected to maintain a consistent computational protocol for the structurally related molecular series and to facilitate a direct comparison of the effects of fluorination and thienyl positional isomerism. However, conventional B3LYP may have limitations for excited states with significant long-range charge-transfer character because of its treatment of the asymptotic exchange potential. Therefore, the TD-B3LYP results obtained in this study are primarily used for comparative evaluation of the designed molecules rather than as quantitatively exact predictions of experimental absorption maxima. Long-range-corrected functionals, such as CAM-B3LYP or ωB97X-type functionals, may provide improved descriptions of long-range charge-transfer excitations and would be useful for future benchmark calculations.

Frontier molecular orbital (HOMO–LUMO) energies were obtained from DFT calculations, while absorption spectra (*λ*_max_) and electronic excitation properties were computed using TD-DFT at the same level of theory. Molecular electrostatic potential (MEP) and global reactivity parameters, including electronegativity, chemical potential, global hardness, global softness, and electrophilicity index, were also evaluated. In addition, noncovalent interactions were analyzed using the reduced density gradient (RDG) approach.

Visualization and post-processing of the computed data were carried out using GaussView 5,^41^ Multiwfn,^42^ and VMD software packages.^43^

## 3. Results and discussions

### 3.1 Geometry optimisation

**Fig. 1:**
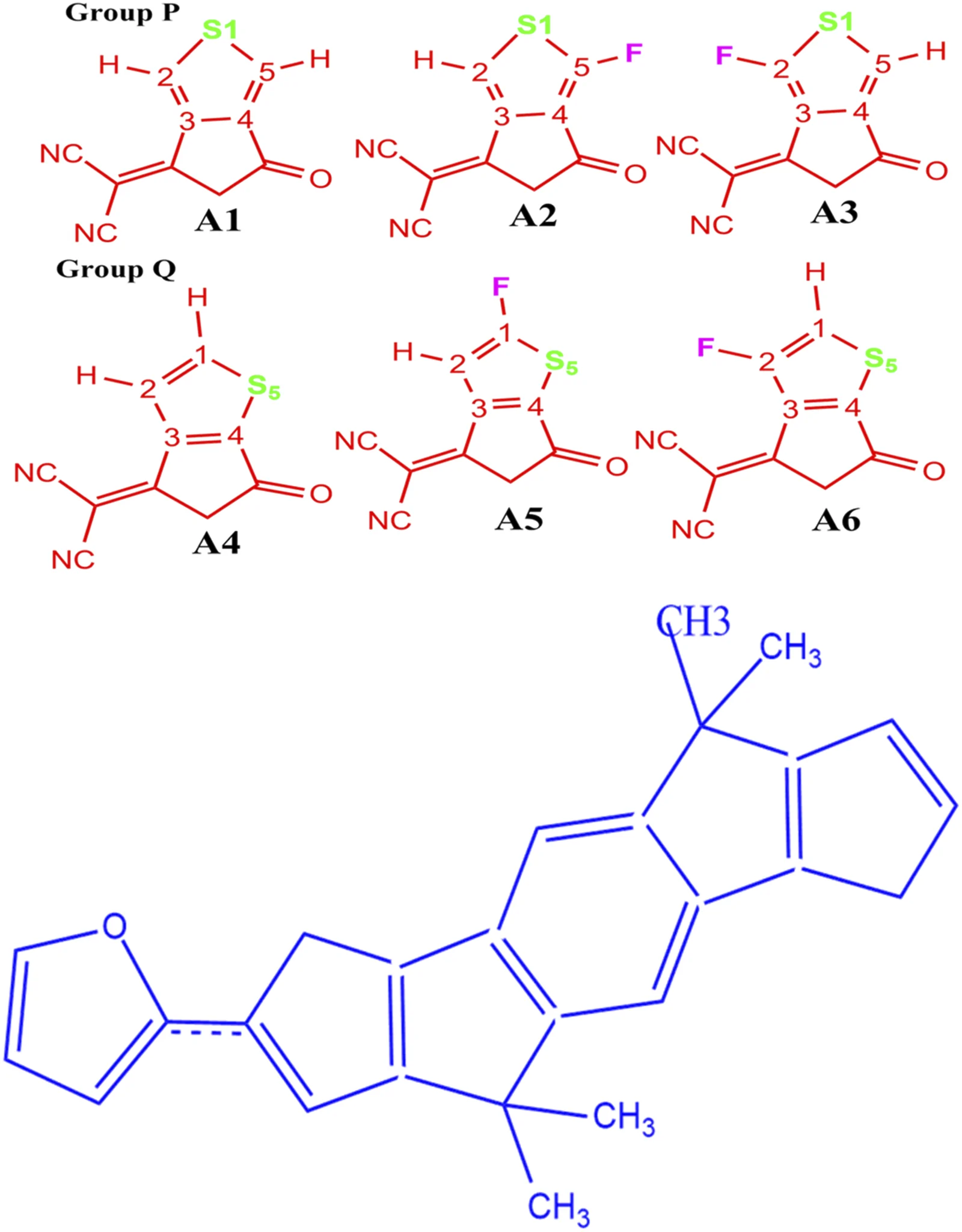
Chemical structures of the designed D–π–A molecules (D1–D6). The acceptor units are highlighted in red, while the donor moiety connected through the furan π-spacer is shown in blue.

A series of six molecules (D1–D6) were designed by systematically varying the positions of fluorine and sulfur atoms within the acceptor units. Initially, the acceptor molecules were optimised and categorised into two groups, namely group P and group Q, based on structural variations. For group P, the reference acceptor (A1) contains a sulfur (S) atom positioned at site 1, as illustrated in Fig. 1. The adjacent carbon atoms at positions 2 and 5 are each bonded to hydrogen atoms. When the hydrogen atom at position 5 is substituted with a fluorine (F) atom, structure A2 is obtained. Similarly, substitution of the hydrogen atom at position 2 with fluorine leads to the formation of another fluorinated derivative. In group Q, the sulfur atom at position 1 is replaced by a carbon atom at position 5, resulting in structure A4. In this configuration, the carbon atoms at positions 1 and 2 are bonded to hydrogen atoms. Further substitution of the hydrogen atom at position 1 with fluorine produces structure A5, while substitution at position 2 yields structure A6. All modified acceptors (A1–A6) are linked to the same donor unit through a furan π-spacer, forming the complete D–π–A architecture.

**Fig. 2:**
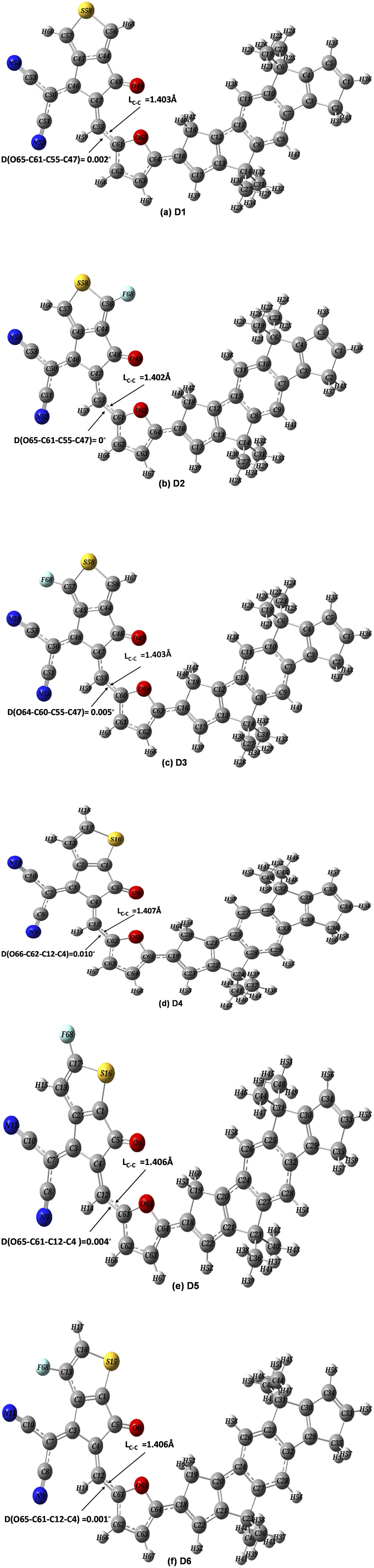
Optimised geometries of the designed molecules (a) D1, (b) D2, (c) D3, (d) D4, (e) D5, and (f) D6 obtained at the B3LYP/6-311G(d,p) level of theory. The bond length (*L*_C–C_) between carbon atoms and the dihedral angle (*θ*) between the acceptor unit and the furan π-spacer are indicated.

The geometry optimisation of all designed molecules was carried out using the B3LYP functional with the 6-311G(d,p) basis set. Optimised structures are presented in Fig. 2, while the corresponding molecular frameworks are shown in Fig. 1, where acceptor units are highlighted in red, and the donor moiety connected *via* the furan π-spacer is shown in blue. The optimised ground-state geometries provide important insights into the optoelectronic behavior of the molecules. Key structural parameters, including the dihedral angle (*θ*) and bond length between the furan spacer and the acceptor unit, were calculated and are summarised in Table 1.

**Table 1:** Optimised bond lengths (*L*_C–C_, Å) and dihedral angles (*θ*^0^) between the furan π-spacer and acceptor units of the designed molecules (D1–D6)

Compounds	Bond length (*L*_C–C_) Å	Dihedral angle *θ* (°)
D1	1.403	0.002
D2	1.402	0.000
D3	1.403	0.005
D4	1.407	0.010
D5	1.406	0.004
D6	1.406	0.001

The optimized molecules are essentially planar, exhibiting negligible dihedral distortions that facilitate effective π-electron delocalization. The calculated bond lengths lie between 1.402 Å and 1.407 Å, which fall between typical single (1.54 Å) and double (1.34 Å) carbon–carbon bond lengths.^44^ This intermediate bond length confirms the presence of partial double-bond character and extended π-conjugation across the molecular backbone. Overall, these structural features indicate strong electronic delocalisation, which is essential for enhancing the optoelectronic performance of the designed molecules (Table 2).

**Table 2:** Calculated HOMO and LUMO energy levels and corresponding energy gaps (*E*_g_) of the designed molecules (D1–D6) in gas and solvent phases (all values in eV)

Molecules	HOMO	LUMO	HOMO (solvent)	LUMO (solvent)	Energy gap	Energy gap (solvent)
D1	−4.8806	−2.9946	−4.8140	−3.0352	1.89	1.78
D2	−4.9027	−3.0507	−4.8306	−3.0942	1.85	1.74
D3	−4.9098	−3.0262	−4.8300	−3.0558	1.88	1.77
D4	−4.8083	−2.8414	−4.7536	−2.9146	1.97	1.84
D5	−4.8610	−2.9347	−4.7612	−2.9748	1.93	1.79
D6	−4.8404	−2.8836	−4.7764	−2.9598	1.96	1.82

### 3.2 Frontier molecular orbitals

**Fig. 3:**
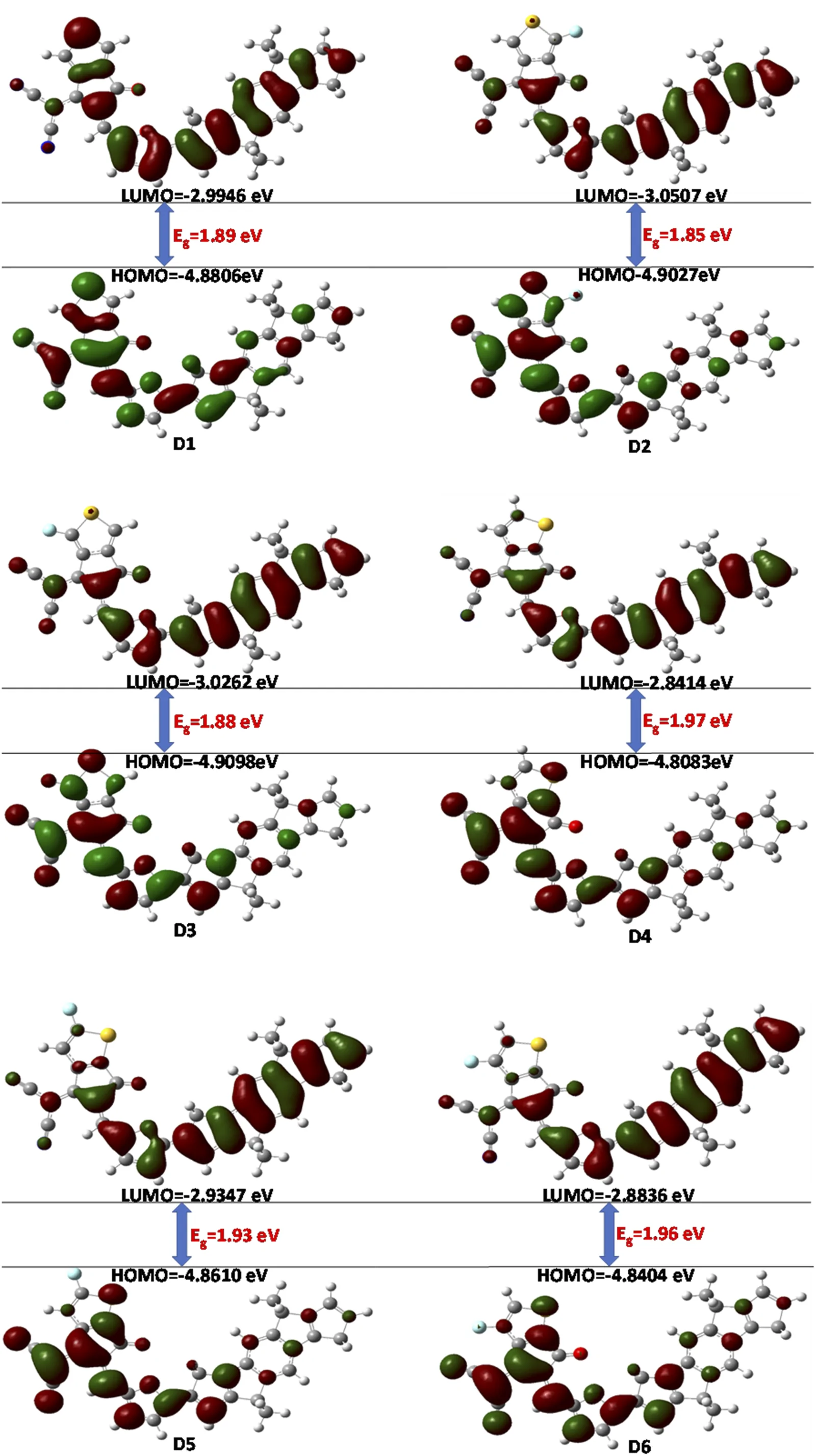
Frontier molecular orbital (FMO) distributions of the designed molecules (D1–D6), showing HOMO and LUMO electron density distributions along with the corresponding energy gaps (*E*_g_).

The energy levels of molecular orbitals play a crucial role in the design of organic solar cells (OSCs), as they directly influence the charge transfer process between donor and acceptor units. Frontier molecular orbitals (FMOs), namely the highest occupied molecular orbital (HOMO) and the lowest unoccupied molecular orbital (LUMO), describe the distribution of electron density in the ground and excited states. Upon photoexcitation, an electron is promoted from the HOMO to the LUMO, initiating the charge transfer process. As illustrated in Fig. 3, the HOMO is predominantly localised over the donor moiety, whereas the LUMO is mainly distributed over the acceptor unit. This spatial separation of electron density facilitates efficient intramolecular charge transfer, which is essential for improved photovoltaic performance. The HOMO–LUMO energy gap (*E*_g_) is a key parameter that governs light absorption, charge mobility, and overall device efficiency.

The FMO diagrams (Fig. 3) also indicate the charge distribution using color mapping, where red represents regions of higher electron density and blue indicates lower electron density. In all designed molecules, the HOMO density is concentrated on the donor unit, while the LUMO density is largely shifted toward the acceptor unit. This clear donor-to-acceptor charge transfer behavior confirms the suitability of these molecules for OSC applications.

The calculated HOMO–LUMO energy gaps of the designed molecules follow the order:D4 (1.97 eV) > D6 (1.96 eV) > D5 (1.93 eV) > D1 (1.89 eV) > D3 (1.86 eV) > D2 (1.85 eV).

All molecules exhibit relatively narrow energy gaps (∼1.85–1.97 eV), which are well within the desirable range for organic photovoltaic materials. A reduced energy gap enhances light absorption and promotes efficient charge transfer. It is observed that modification of sulfur position results in an energy gap variation of approximately 0.08 eV, while additional fluorine substitution further decreases the band gap by about 0.12 eV, indicating improved electronic tuning. In the presence of chlorobenzene solvent, slight variations in orbital energies are observed. The HOMO energy levels increase (become less negative), ranging from −4.75 eV to −4.83 eV, indicating a slight destabilisation due to solvent polarisation effects. In contrast, the LUMO energy levels decrease (become more negative), ranging from −2.95 eV to −3.09 eV, suggesting stabilisation of the acceptor levels.

Consequently, the HOMO–LUMO energy gaps in the solvent phase are reduced and follow the order:D4 (1.84 eV) > D6 (1.82 eV) > D5 (1.79 eV) > D1 (1.78 eV) > D3 (1.77 eV) > D2 (1.74 eV).

The overall reduction in energy gap (∼0.1 eV) upon solvent inclusion indicates a red shift in absorption, which enhances light-harvesting capability. This decrease in band gap also suggests improved intermolecular charge transfer and lower excitation energy, both of which are favorable for optoelectronic and photovoltaic applications. Therefore, the solvent environment plays a significant role in tuning the electronic structure and improving the performance of the designed OSC materials.

### 3.3 Global chemical reactivity parameters (GCRP)

The global chemical reactivity of the designed molecules was evaluated using quantum chemical descriptors derived from frontier molecular orbital energies. These parameters provide valuable insight into the stability, reactivity, and charge transfer characteristics of the molecules. Natural bond orbital (NBO) analysis, along with Koopmans' theorem, was employed to estimate key reactivity descriptors based on HOMO and LUMO energies.^45,46^

The following equations were used to calculate the global reactivity parameters:

1 Ionization potential IP = −*E*_HOMO_

2 Electron affinity EA = −*E*_LUMO_

3
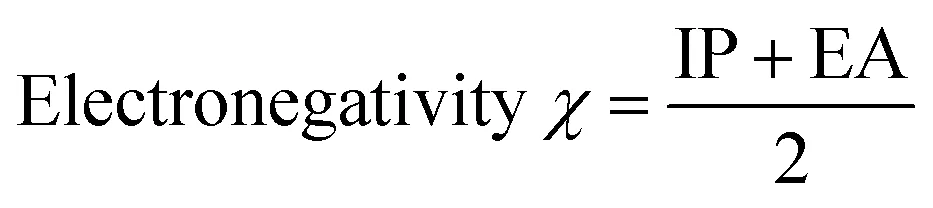

4 Chemical potential *µ* = −*χ*

5
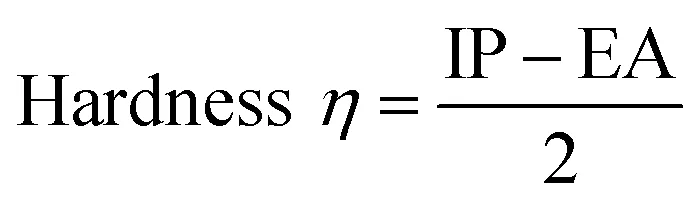
6
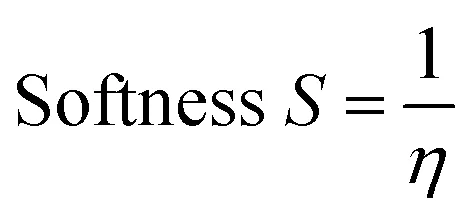
7
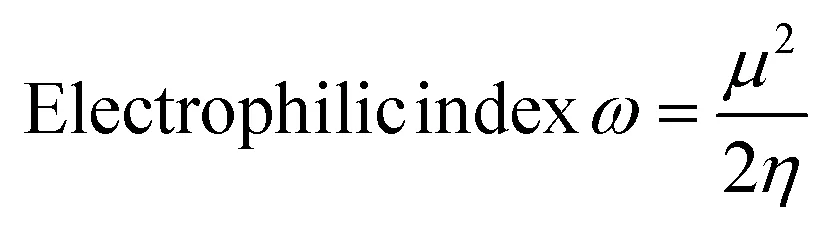


The calculated values of these parameters for all molecules (D1–D6) are summarized in Table 3. Ionization potential (IP) and electron affinity (EA) are key indicators of molecular reactivity and electronic structure. Among the studied compounds, molecules in group P exhibit relatively higher electron affinity values, following the trend: D2 (3.05 eV) > D3 (3.03 eV) > D1 (2.99 eV). Higher EA values indicate a stronger ability to accept electrons, which enhances charge transfer efficiency.

**Table 3:** Global chemical reactivity parameters of the designed molecules (D1–D6), including ionization potential (IP), electron affinity (EA), electronegativity, chemical potential, global hardness, global softness, electrophilicity index, and dipole moments in gas and solvent phases

Molecule	IP	EA	Electro- negativity (χ)	Chemical potential(*µ*)	Global hardness (*η*)	Global softness (*S*)	Electrophilicity index (*ω*)	Dipole moment (in Debye)	Dipole moment in solvent (in Debye)
D1	4.8806	2.9946	3.9400	−3.9400	0.9400	0.5300	8.2200	12.7002	16.4050
D2	4.9027	3.0507	3.9800	−3.9800	0.9300	0.5400	8.5400	12.3465	15.2706
D3	4.9098	3.0262	3.9700	−3.9700	0.9400	0.5300	8.3600	13.9741	15.9630
D4	4.8083	2.8414	3.8200	−3.8200	0.9800	0.5100	7.4400	10.7878	13.4541
D5	4.8610	2.9347	3.9000	−3.9000	0.9600	0.5200	7.8900	11.3548	14.0813
D6	4.8404	2.8836	3.8600	−3.8600	0.9800	0.5100	7.6200	12.3192	15.5068

Global softness (*S*) is associated with the ease of charge redistribution within a molecule. Molecule D2 shows the highest softness value (0.54), suggesting enhanced charge mobility and improved charge separation characteristics. In contrast, global hardness (*η*), which reflects resistance to charge transfer, is lowest for D2 (0.93) and D5 (0.94), indicating that these molecules are more reactive and better suited for photovoltaic applications.

The electrophilicity index (*ω*) describes the electron-accepting capability of a molecule. Among all compounds, D2 exhibits the highest electrophilicity value (8.54), indicating a strong tendency to accept electrons and contribute to higher photocurrent generation. Dipole moment is another important parameter that reflects molecular polarity and stability.^47^ Molecules in group P generally show higher dipole moments compared to group Q, indicating stronger intermolecular interactions. Notably, D3 exhibits the highest dipole moment (13.97 Debye) in the gas phase.

Upon inclusion of solvent effects, the dipole moment increases by approximately 3 Debye for all molecules. This enhancement can be attributed to solvent – solute interactions, which stabilize the polarized electronic distribution and promote greater charge separation. The increased dipole moment in the solvent phase suggests a strengthened intramolecular charge transfer (ICT) character, which is highly beneficial for optoelectronic and photovoltaic performance.

### 3.4 Density of states (DOS)

**Fig. 4:**
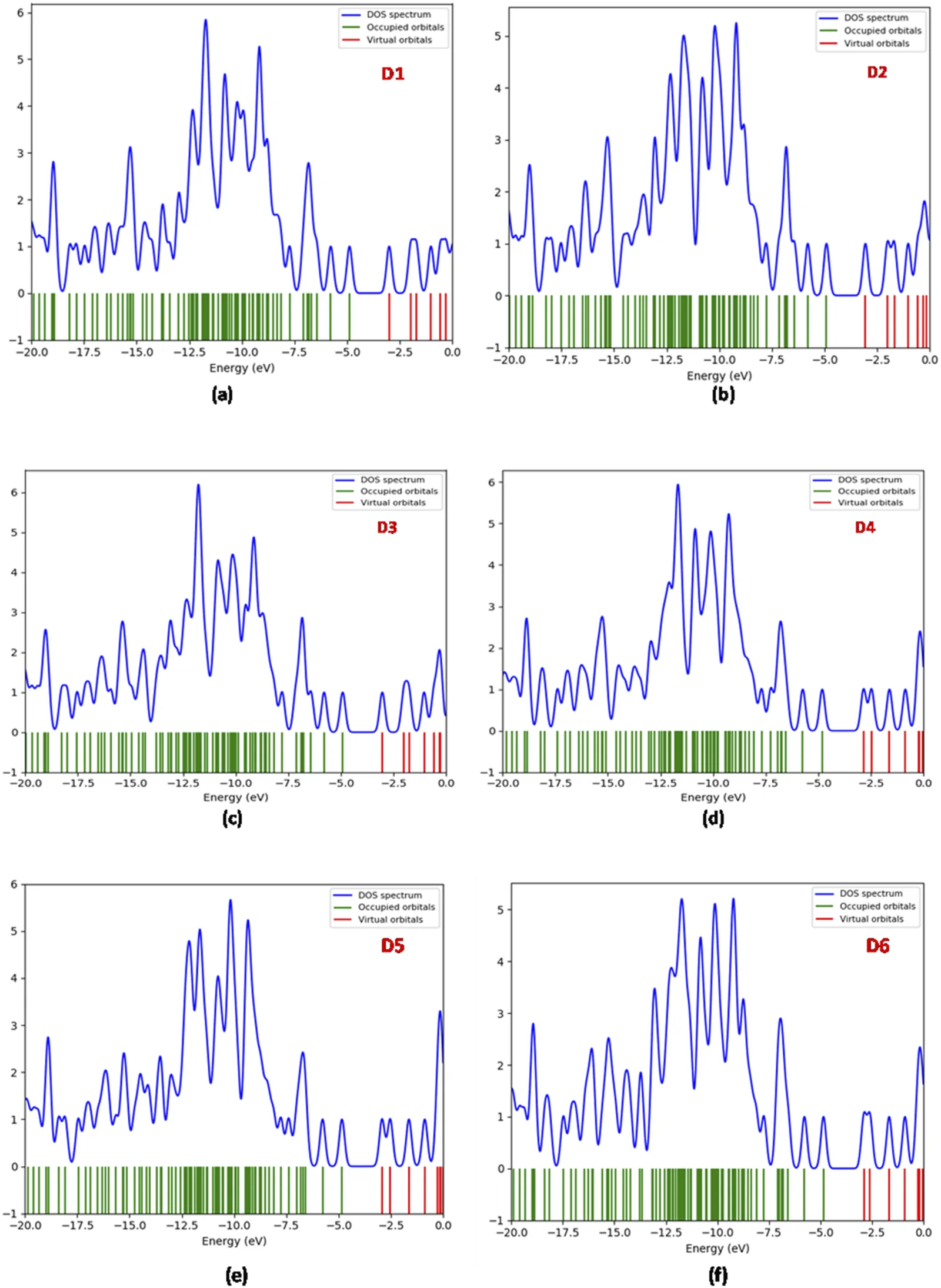
Density of states (DOS) profiles of the designed molecules (a) D1, (b) D2, (c) D3, (d) D4, (e) D5, and (f) D6, illustrating the contribution of donor and acceptor units to the electronic structure.

Density of states (DOS) analysis provides detailed insight into the distribution of electronic states across different energy levels, particularly around the HOMO and LUMO regions. It is an important tool for understanding the electronic structure, charge transfer behavior, and recombination processes in organic solar cell materials. The DOS profile also reflects the availability of electronic states for excitation and charge transport. The DOS spectra of all designed molecules (D1–D6), obtained using GaussSum software,^48^ are presented in Fig. 4. In the DOS plots, the separation between the donor and acceptor contributions indicates the energy required for electron excitation from the donor (HOMO) to the acceptor (LUMO). A smaller separation corresponds to a lower excitation energy, which is beneficial for efficient charge transfer.

The results reveal that fluorine-substituted molecules (D2, D3, D5, and D6) exhibit reduced energy separation compared to the non-substituted molecules (D1 and D4). This indicates that fluorination facilitates easier electron excitation and enhances charge transfer efficiency. Additionally, variations in the DOS profiles among the molecules suggest that structural modifications significantly influence their electronic properties. Overall, the DOS analysis supports the improved optoelectronic performance of the fluorinated compounds.

### 3.5 Molecular electrostatic potential (MEP)

Molecular electrostatic potential (MEP) analysis is a valuable tool for identifying reactive sites within a molecule and understanding its interaction with other chemical species.^49^ The MEP surface represents the distribution of electrostatic potential over the molecular surface, which is closely related to electron density. In the MEP maps, different colors correspond to different electrostatic potentials: red regions indicate areas of high electron density (negative potential), blue regions represent electron-deficient areas (positive potential), and green/yellow regions correspond to nearly neutral potential. These color variations help in identifying nucleophilic and electrophilic regions within the molecule.

**Fig. 5:**
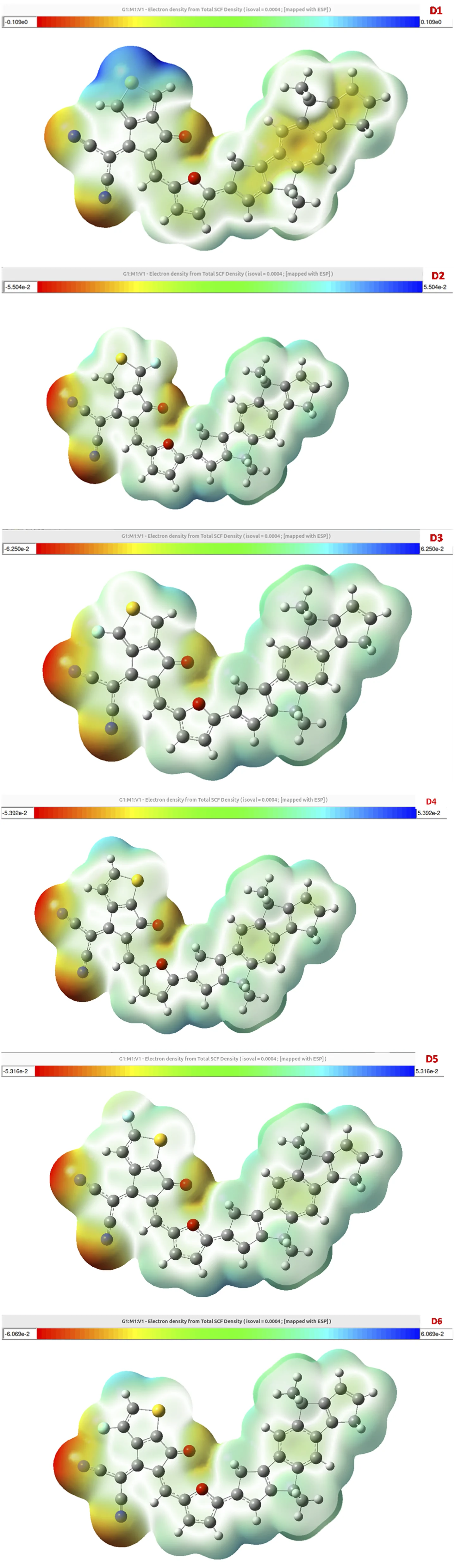
Molecular electrostatic potential (MEP) surfaces of the designed molecules (D1–D6), indicating regions of electron-rich (red), electron-deficient (blue), and neutral (green/yellow) electrostatic potential.

The MEP surfaces of all designed molecules were computed at the B3LYP/6-311G(d,p) level of theory and are shown in Fig. 5. The results indicate that electron density is predominantly concentrated around the acceptor units, particularly near electronegative atoms such as oxygen and nitrogen. In contrast, the donor regions exhibit relatively lower electron density. This distribution suggests that the acceptor regions are more favorable for electrophilic interactions, while the donor regions are more prone to nucleophilic interactions. The clear separation of electron-rich and electron-deficient regions supports efficient intramolecular charge transfer (ICT).

### 3.6 Optical properties (UV-visible analysis)

**Fig. 6:**
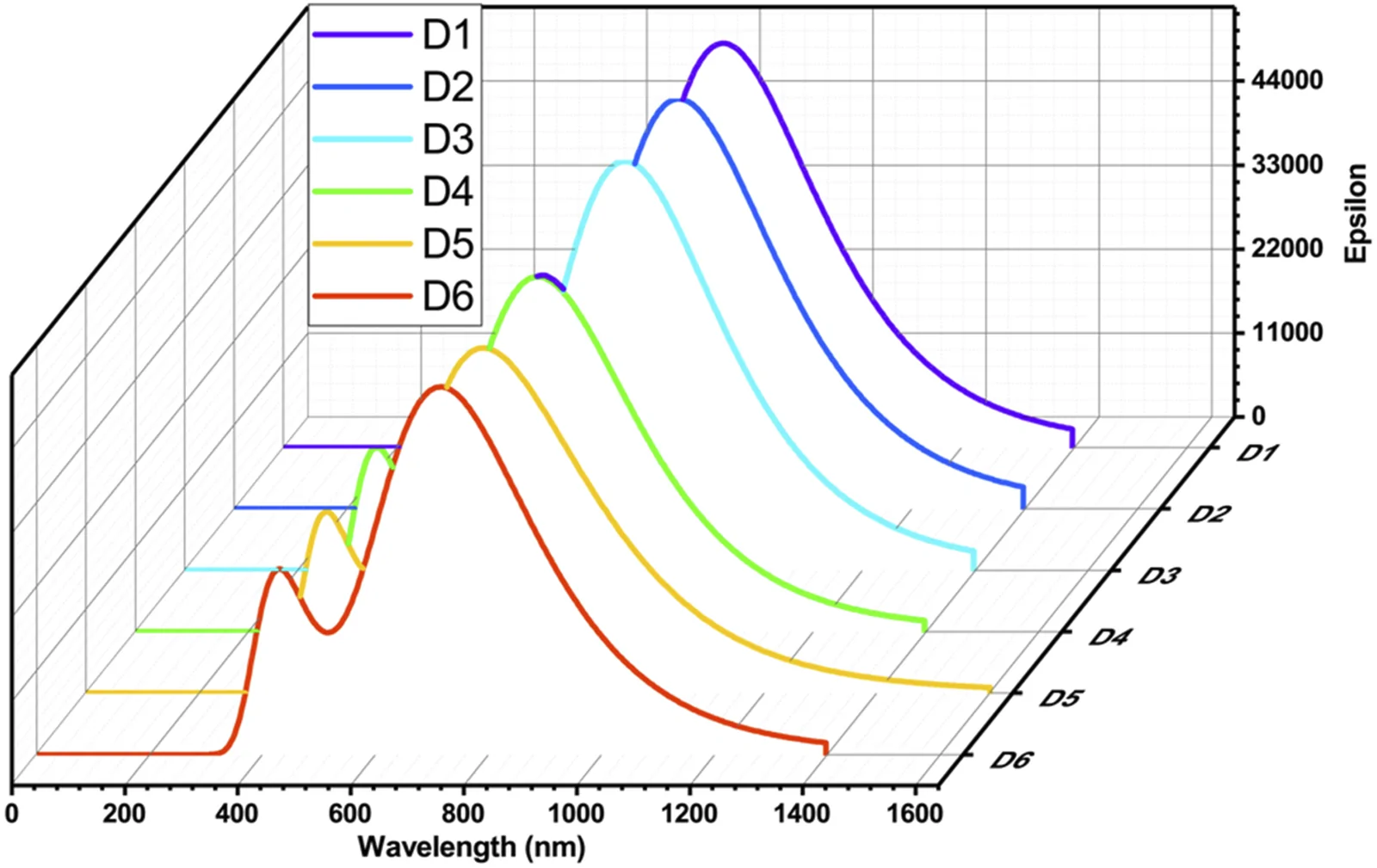
Simulated UV-visible absorption spectra of the designed molecules (D1–D6), showing the maximum absorption wavelength (*λ*_max_).

Efficient conversion of light into electrical energy in organic solar cells requires strong absorption in the visible and near-infrared (NIR) regions of the solar spectrum. Therefore, UV-visible analysis is a crucial parameter for evaluating the suitability of designed molecules for photovoltaic applications.^50^ The electronic excitation properties of the molecules were investigated using time-dependent density functional theory (TD-DFT) at the B3LYP/6-311G(d,p) level of theory. The simulated absorption spectra of all designed molecules (D1–D6) are presented in Fig. 6, while the corresponding optical parameters, including maximum absorption wavelength (*λ*_max_), excitation energy (*E*_ex_), oscillator strength (*f*), and major electronic transitions, are summarised in Table 4.

**Table 4:** Simulated optical properties of the designed molecules (D1–D6), including maximum absorption wavelength (*λ*_max_, nm), excitation energy (Δ*E*, eV), oscillator strength (*f*), and major electronic transitions

Molecule name	State	*λ*_max_ (nm)	Δ*E* (eV)	Oscillation strength (*f*)	Transition
D1	S1	780.13	1.58	1.3018	HOMO → LUMO (100%)
	S2	522.99	2.37	0.2239	HOMO → LUMO+1(60.7%)
	S3	501.22	2.47	0.1262	HOMO −1 → LUMO (50.1%)
D2	S1	789.02	1.57	1.3203	HOMO → LUMO (100%)
	S2	526.39	2.35	0.2669	HOMO −1 → LUMO (50.8%)
	S3	498.65	2.48	0.0923	HOMO → LUMO +1(49.5%)
D3	S1	781.31	1.58	1.3180	HOMO → LUMO(100%)
	S2	524.22	2.36	0.1997	HOMO −1 → LUMO (59.7%)
	S3	502.93	2.46	0.1306	HOMO −1 → LUMO (49.1%)
D4	S1	788.47	1.57	0.6415	HOMO → LUMO (94.8%)
	S2	659.35	1.88	0.6817	HOMO → LUMO+1 (94.2%)
	S3	501.22	2.47	0.1262	HOMO −1 → LUMO (88.5%)
D5	S1	820.05	1.51	0.5278	HOMO → LUMO (94.9%)
	S2	661.91	1.87	0.8076	HOMO → LUMO +1 (94.6%)
	S3	519.4	2.38	0.1255	HOMO −1 → LUMO (89.9%)
D6	S1	805.36	1.53	0.4987	HOMO → LUMO (92.7%)
	S2	680.12	1.82	0.8270	HOMO → LUMO +1(92.6%)
	S3	511.31	2.42	0.1436	HOMO −1 → LUMO (89.2%)

All molecules exhibit broad absorption in the visible to near-infrared region, ranging from approximately 400 nm to 950 nm, indicating their strong light-harvesting capability. The dominant electronic transition in all cases corresponds primarily to HOMO → LUMO excitation. For group P molecules (D1–D3), the HOMO–LUMO contribution reaches nearly 100%, indicating a well-defined and efficient excitation process. In contrast, group Q molecules (D4–D6) show slightly lower contributions (∼94%), suggesting minor involvement of additional orbitals.

Among all compounds, the fluorine-substituted molecule D2 exhibits the highest oscillator strength (*f* = 1.3203), indicating a strong probability of electronic transition and efficient photon absorption at its peak wavelength in the solvent phase (Table 4).

The maximum absorption wavelengths (*λ*_max_) in chlorobenzene solvent follow the order:D5 (820.05 nm) > D6 (805.36 nm) > D2 (789.02 nm) > D4 (788.47 nm) > D3 (781.31 nm) > D1 (780.13 nm).

This trend demonstrates that structural modification, particularly fluorine substitution, induces a red shift in absorption, thereby enhancing light absorption in the NIR region. Compared to the reference molecules (D1 and D4), the modified compounds exhibit improved absorption characteristics.

### 3.7 Open circuit voltage (*V*_oc_) and fill factor analysis

The performance of organic solar cells (OSCs) is strongly influenced by the open-circuit voltage (*V*_oc_), which represents the maximum voltage obtained under zero-current conditions.^51^ It is a key parameter that determines the overall power conversion efficiency (PCE) of the device.

The open-circuit voltage can be estimated using the Scharber equation:^52^
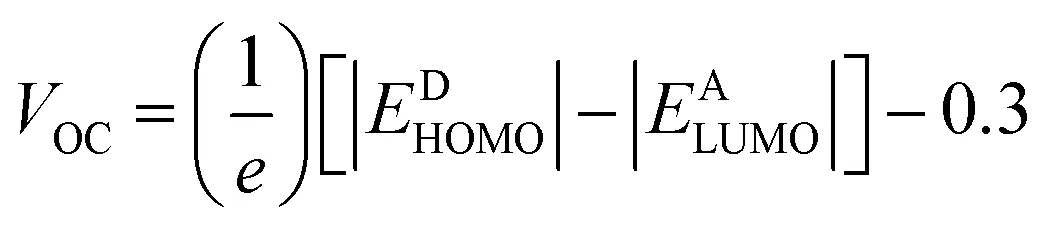
where *E*^D^_HOMO_ is the HOMO energy of the donor material (here, P3HT), *E*^A^_LUMO_is the LUMO energy of the acceptor molecules, *e* is the elementary charge, and 0.3 eV accounts for empirical energy losses in the device.^53^

For the reference donor polymer P3HT, the HOMO and LUMO energy levels are −5.14 eV and −3.14 eV, respectively.^54^ The LUMO energy levels of all designed acceptor molecules are lower than that of P3HT, ensuring efficient electron transfer from the donor to the acceptor.

The calculated *V*_oc_ values for the designed molecules follow the order:D4 (1.47 V) > D1 (1.39 V) > D6 (1.37 V) > D3 (1.36 V) > D5 (1.33 V) > D2 (1.27 V).

Among all molecules, D4 exhibits the highest *V*_oc_ value, indicating its strong potential for photovoltaic applications. The favorable alignment of HOMO and LUMO energy levels confirms efficient charge separation and transfer within the system (Table 5).

**Table 5:** Calculated open-circuit voltage (*V*_oc_), fill factor (FF), and percentage fill factor (%FF) of the designed molecules (D1–D6) using P3HT as the reference donor

Molecule name	*E* ^D^_HOMO_ (eV)	*E* ^A^_LUMO_ (eV)	*V*_oc_(V)	FF	%FF
D1	−5.14	−3.450	1.39	0.8912	89.12
D2	−5.14	−3.568	1.27	0.8764	87.64
D3	−5.14	−3.485	1.36	0.8889	88.89
D4	−5.14	−3.371	1.47	0.9006	90.06
D5	−5.14	−3.506	1.33	0.8845	88.45
D6	−5.14	−3.475	1.37	0.8898	88.98

In addition to *V*_oc_, the fill factor (FF) is another important parameter that contributes to the overall efficiency of OSCs. The FF was estimated using the following empirical relation based on *V*_oc_:^55^
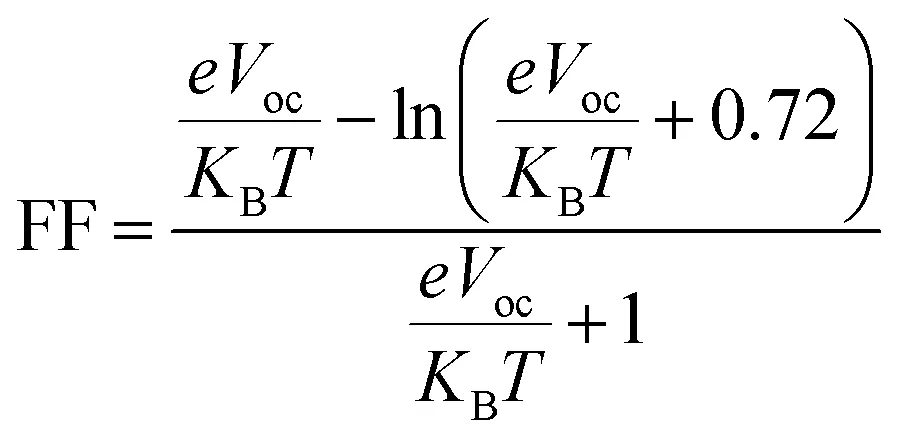
where *K*_B_ is the Boltzmann constant, *T* is the temperature (300 K), and *e* is the elementary charge.

The calculated FF values range from 0.8764 to 0.9006, which are close to the theoretical limit for OSCs. Such high values are attributed to the idealised conditions of the model, where factors such as interfacial recombination, series resistance, shunt losses, and morphological disorder are not considered.

The FF values follow the order:D4 (0.9006) > D1 (0.8912) > D6 (0.8898) > D3 (0.8889) > D5 (0.8845) > D2 (0.8764).

**Fig. 7:**
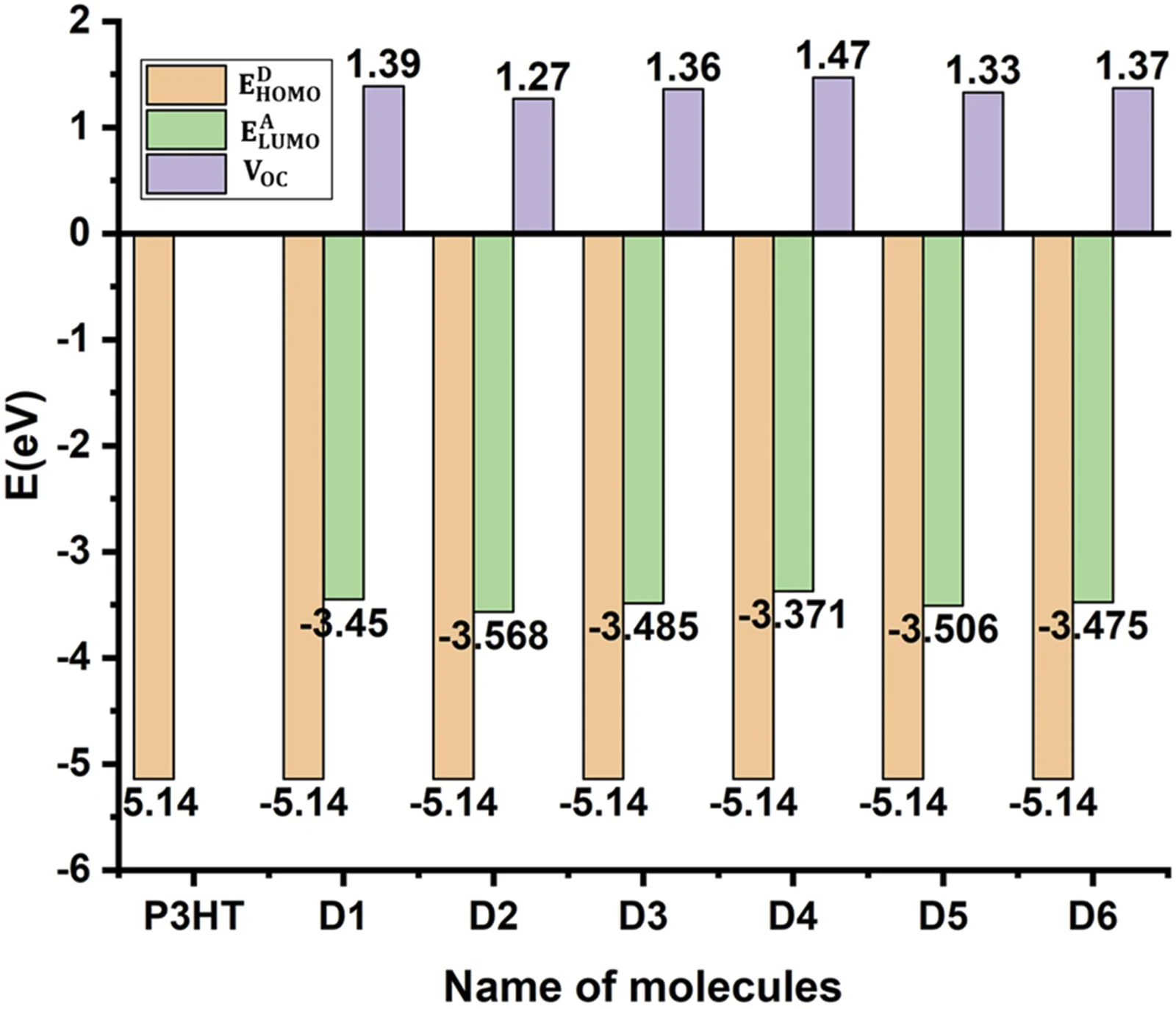
Open-circuit voltage (*V*_oc_) of the designed molecules (D1–D6), calculated using the HOMO energy of the donor (P3HT) and LUMO energies of the acceptor molecules.

Higher FF values indicate more efficient charge extraction and reduced energy losses, suggesting that the designed molecules possess promising characteristics for high-performance organic solar cells (Fig. 7).

#### 3.7.1 Photovoltaic parameters

The open-circuit voltage (*V*_oc_) of the designed acceptors was assessed utilizing a Scharber-type methodology, with P3HT serving as the reference donor. This choice of P3HT as a standard reference system facilitates a uniform assessment of the energetic compatibility among D1–D6. Nevertheless, it is important to note that P3HT may not accurately reflect the donor materials used in many of the leading contemporary non-fullerene acceptor (NFA)-based organic solar cells (OSCs). As such, the calculated *V*_oc_ values should be regarded as theoretical molecular screening indicators rather than direct forecasts of voltages achievable in experimental devices.

The determined *V*_oc_ values fall within the range of 1.27 to 1.47 V, with D4 exhibiting the highest estimated value. The variations in these values primarily stem from differences in the LUMO energies of the acceptor molecules. However, it is also critical to recognize that the actual *V*_oc_ of an OSC is affected by factors such as interfacial energetics, non-radiative voltage losses, charge recombination processes, morphology, and energetic disorder.

In this study, *J*_sc_ and power conversion efficiency (PCE) were not evaluated because accurate predictions for these parameters necessitate additional data on photon absorption rates, exciton generation occurrences, charge-transfer efficiencies, carrier mobilities, recombination dynamics, molecular packing arrangements, blend morphology characteristics, and device architecture specifics. Therefore, deriving numerical PCE or *J*_sc_ values exclusively from isolated-molecule density functional theory (DFT) calculations would introduce considerable uncertainty. Consequently, the findings presented here should be interpreted as results from molecular-level screening that require confirmation through subsequent device fabrication and characterization efforts.

### 3.8 Exciton binding energy (*E*_b_)

Exciton binding energy (*E*_b_) serves as a crucial metric for assessing the viability of organic semiconductor materials in photovoltaic applications. When sunlight is absorbed, an electron transitions from a filled to an empty molecular orbital, resulting in the formation of a coulombically bound electron–hole pair, known as an exciton. In order for photocurrent generation to occur within an organic solar cell, this exciton must then dissociate into more spatially separated charge carriers, typically at the interface between donor and acceptor materials. Consequently, the level of exciton binding energy offers valuable insights into the energy requirements necessary for the separation of electron–hole pairs.

Exciton binding energy (*E*_b_) is defined as the energy required to separate a bound electron–hole pair (exciton) into free charge carriers.^56^ It is an important parameter that influences charge separation efficiency and, consequently, the photovoltaic performance of the material.

The exciton binding energy was calculated using the relation:^57^*E*_b_ = *E*_g_ − *E*_opt_where *E*_g_ is the HOMO–LUMO energy gap and *E*_opt_ is the excitation energy corresponding to the transition from the ground state (S_0_) to the first excited state (S_1_). This concept is based on the Wannier–Mott exciton model.^58^

**Fig. 8:**
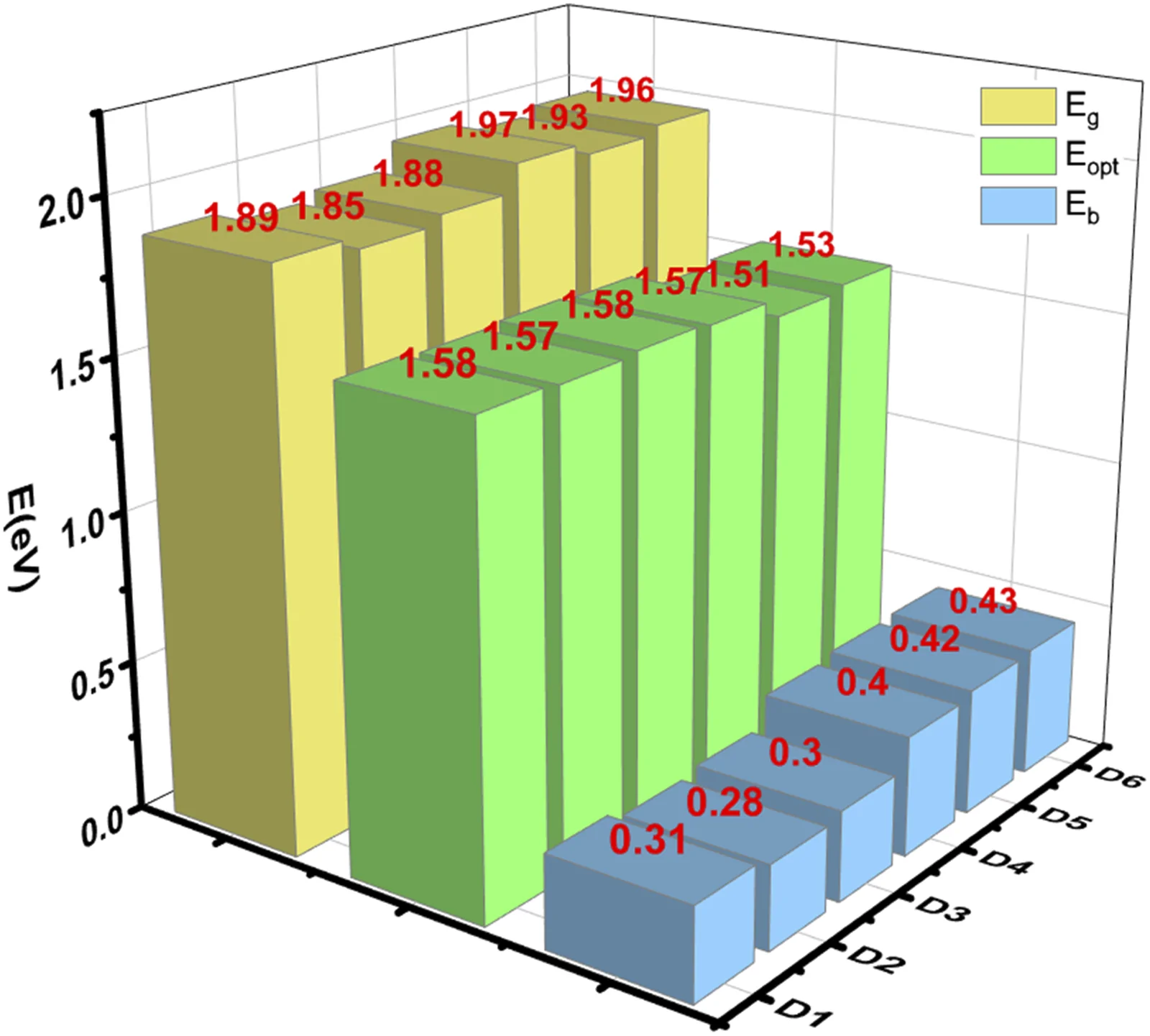
Comparison of energy gap (*E*_g_), excitation energy (*E*_opt_), and exciton binding energy (*E*_b_) of the designed molecules (D1–D6).

The calculated *E*_b_ values for D1–D6 are 0.31, 0.28, 0.30, 0.40, 0.42, and 0.43 eV, respectively. Thus, D2 exhibits the lowest exciton binding energy (0.28 eV), followed by D3 (0.30 eV) and D1 (0.31 eV), whereas D6 exhibits the highest value (0.43 eV) (Table 6). These calculated values are summarized and compared with the corresponding electronic and optical gaps in Fig. 8.

**Table 6:** Exciton binding energy (*E*_b_) of the designed molecules (D1–D6), calculated from the difference between the electronic band gap (*E*_g_) and optical excitation energy (*E*_opt_)

Molecule name	Energy gap (in eV)	*E*_opt_ (eV)	*E*_b_(eV)
D1	1.89	1.58	0.31
D2	1.85	1.57	0.28
D3	1.88	1.58	0.3
D4	1.97	1.57	0.4
D5	1.93	1.51	0.42
D6	1.96	1.53	0.43

The comparatively lower *E*_b_ of D2 indicates a diminished coulombic attraction between electrons and holes, leading to a reduced energy barrier for exciton dissociation. This feature is advantageous for organic photovoltaic systems since more efficient exciton dissociation enhances the likelihood of producing free charge carriers, which can result in increased photocurrent and decreased geminate recombination. Thus, the low *E*_b_ associated with D2, along with its relatively narrow HOMO–LUMO gap and pronounced low-energy optical transition, positions D2 as a promising candidate for further exploration as a non-fullerene acceptor (NFA) material.

However, it is important to note that exciton binding energy should not be viewed as an isolated indicator of power conversion efficiency (PCE). In practical organic solar cell (OSC) applications, exciton dissociation is significantly affected by factors such as the alignment of donor–acceptor energy levels, the energetics of charge-transfer states, dielectric screening effects, molecular arrangement, blend morphology, intermolecular interactions, charge-carrier mobility, and both geminate and nongeminate recombination processes. Therefore, while a lower calculated *E*_b_ suggests favorable molecular characteristics at the microscopic level, it does not inherently guarantee enhancements in short-circuit current density (*J*_sc_), fill factor, or PCE. To ascertain whether the anticipated beneficial exciton properties of D2 result in improved photovoltaic performance, experimental studies involving thin films and devices are necessary.

### 3.9 Reduced density gradient (RDG) and non-covalent interaction (NCI) analysis

**Fig. 9:**
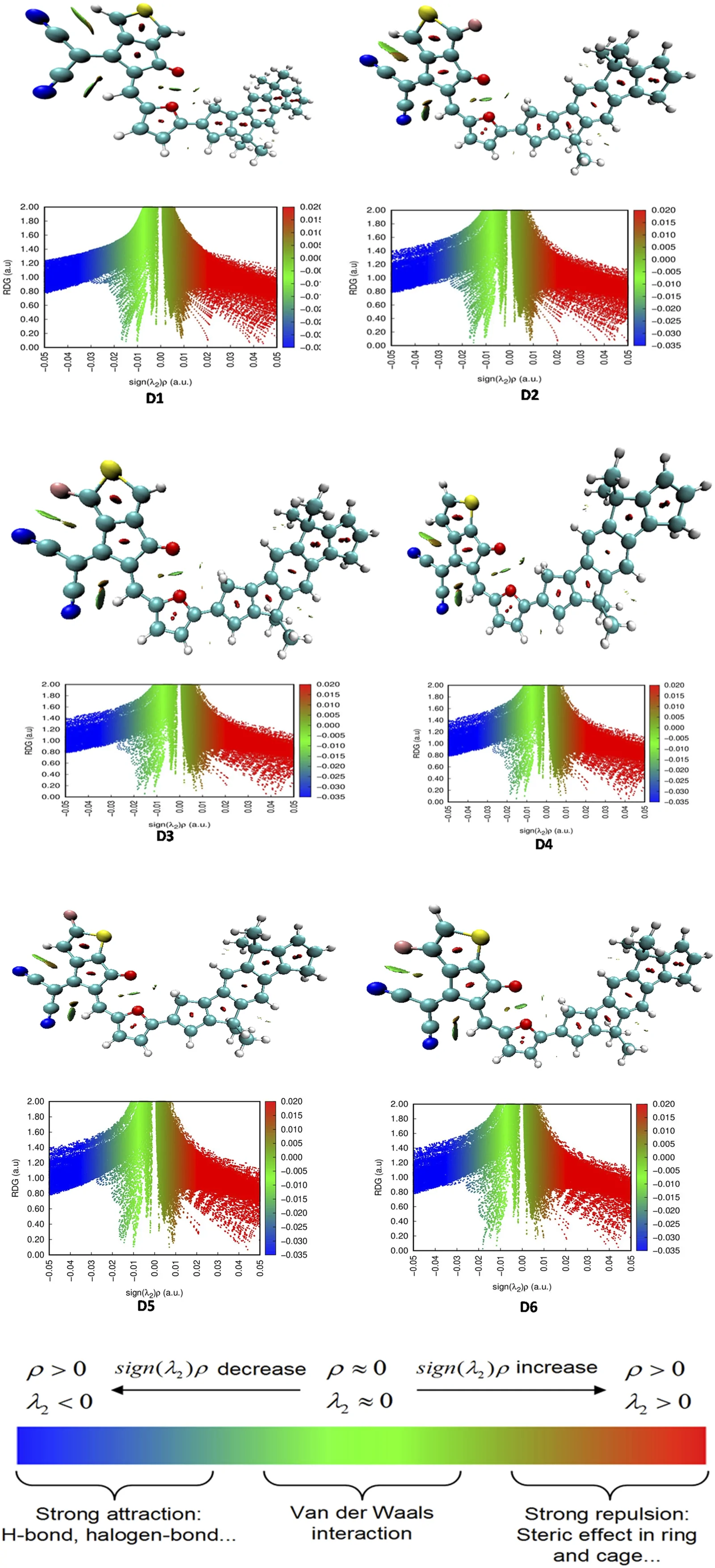
Reduced density gradient (RDG) iso-surfaces and non-covalent interaction (NCI) analysis of the designed molecules (D1–D6), illustrating attractive, weak van der Waals, and repulsive interactions.

The reduced density gradient-non-covalent interaction (RDG-NCI) analysis was conducted to discern and illustrate the characteristics and spatial arrangement of non-covalent interactions within the synthesized D1–D6 molecules. The resultant RDG-NCI isosurfaces are depicted in Fig. 9. The interaction areas were analyzed using the sign(*λ*_2_)*ρ* function, which facilitates the differentiation of attractive, weakly interacting, and repulsive regions based on their color coding.^59^

As illustrated in Fig. 9, blue regions correspond to negative values of sign(*λ*_2_)*ρ*, indicating attractive interactions associated with stronger contacts within the molecular structure. Green regions, located around sign(*λ*_2_)*ρ* ≈ 0, signify weak non-covalent interactions primarily influenced by van der Waals and dispersion forces. Such minor interactions play a crucial role in π-conjugated organic semiconductor molecules because they can affect stability and intermolecular arrangement in conjugated systems. In contrast, red regions indicate positive values of sign(*λ*_2_)*ρ*, reflecting sterically repulsive interactions linked to overlapping electron densities or molecular crowding.

The RDG-NCI surfaces for D1–D6 presented in Fig. 9 exhibit a mix of attractive, weak dispersive, and repulsive interaction zones. The variation in distribution and intensity of these colored areas among the designed molecules stems from differing fluorination patterns and thienyl positions within the terminal acceptor units. Specifically, fluorine substitution alters the local electron-density distribution of the acceptor moiety, thereby influencing the spatial arrangement of non-covalent interaction features. The presence of green regions across the analyzed molecules indicates that weak dispersion-type interactions significantly contribute to their non-covalent interaction profile; conversely, localized blue and red areas represent stronger attractive contacts and steric repulsion.

Notably, observations from Fig. 9 reveal that modifications to terminal groups can shift the equilibrium between attractive and repulsive interactions within these molecular systems. However, it is essential to note that since the current RDG-NCI calculations were carried out on isolated optimized molecules, these observed interaction regions should not be construed as definitive evidence of solid-state π–π stacking or crystallinity nor thin-film morphology. To accurately determine intermolecular packing and morphology for these acceptors would necessitate experimental thin-film characterization or periodic solid-state calculations.

## 4. Conclusion

In this study, a series of six novel donor–π–acceptor (D–π–A) molecules (D1–D6) based on 1,1,6,6-tetramethyl-*as*-indacene as the donor, furan as the π-spacer, and thienyl-fused IC derivatives as acceptors were systematically designed and investigated using DFT and TD-DFT approaches. Structural modification through positional variation of sulfur and fluorine substitution proved to be an effective strategy for tuning the optoelectronic properties of the molecules. All designed compounds exhibited nearly planar geometries, facilitating strong π-conjugation and efficient intramolecular charge transfer. Frontier molecular orbital analysis confirmed clear spatial separation between HOMO and LUMO densities, promoting effective donor-to-acceptor charge transfer. The calculated energy gaps (1.85–1.97 eV in gas phase and further reduced in solvent) fall within the optimal range for organic photovoltaic applications, with solvent effects inducing a noticeable red shift in absorption.

Optical analysis revealed broad absorption spanning the visible to near-infrared region (400–950 nm), with fluorine-substituted molecules showing enhanced oscillator strength and red-shifted *λ*_max_ values. Global reactivity descriptors indicated improved electronic softness, electrophilicity, and dipole moments, particularly for molecule D2, suggesting superior charge transfer characteristics and molecular stability. Furthermore, density of states (DOS), molecular electrostatic potential (MEP), and RDG-NCI analyses confirmed favorable charge distribution, well-defined reactive sites, and the presence of stabilising non-covalent interactions. Photovoltaic parameters demonstrated promising performance, with high open-circuit voltages (up to 1.47 V), excellent fill factors (∼0.88–0.90), and low exciton binding energies (0.28–0.43 eV), indicating efficient exciton dissociation and charge transport.

Among the studied molecules, D2 and D5 emerged as the most promising candidates due to their optimal balance of low energy gap, strong light absorption, high oscillator strength, and reduced exciton binding energy. Overall, this work highlights the effectiveness of end-group engineering and fluorine substitution in enhancing the performance of non-fullerene acceptors. These findings provide valuable design insights for next-generation organic solar cell materials. Future experimental validation and device-level investigations are recommended to further explore the practical applicability and efficiency of the proposed molecular systems.

## Conflicts of interest

The authors declare no competing interests.

## Data Availability

All the data for this study are provided in the manuscript.
